# Agricultural irrigation development in Castilla y León (Spain): driving forces and outcomes for landscape and sustainability in the 21st century

**DOI:** 10.1007/s10980-024-01977-y

**Published:** 2024-11-07

**Authors:** Fabienne Frey, Franziska Mohr, Virginia Ruiz‐Aragón, Felicia O. Akinyemi, Matthias Bürgi

**Affiliations:** 1 Land Change Science Research Unit, Swiss Federal Research Institute WSL, Birmensdorf, Switzerland; 2 Department of Environmental and Biological Sciences, Karlstad University, Karlstad, Sweden; 3 Institute of Geography, University of Bern, Bern, Switzerland

**Keywords:** Irrigation modernization, Sustainable intensification, Land consolidation, Mixed-methods, Climate change

## Abstract

**Context:**

Agriculture relies on irrigation in many parts of the world, and the need for irrigation is increasing due to rising demands for agricultural products and climate change-induced alterations in rainfall patterns. However, irrigated agriculture has been found to damage ecosystems and threaten landscape sustainability.

**Objectives:**

Against this background, there has been a recent development towards large-scale irrigation in Spain. The aim of this study is to understand this development at the landscape level and its impact in the context of landscape sustainability.

**Methods:**

We focused on two study sites in Castilla y León using a mixed-methods approach. We studied driving forces, landscape changes, and sustainability outcomes through document analysis, interviews, and aerial photograph analysis.

**Results:**

The development of a landscape-level underground pipe network took place at one study site and is planned for the second study site. Interviewees perceived institutional and social driving forces as particularly influential and technological driving forces as less influential. Political and economic driving forces were often interlinked. The irrigation development tied to land consolidation led to landscape changes, such as the removal of trees and increases in field size. Thus, in terms of environmental sustainability, trade-offs were found, while social sustainability outcomes were mainly positive. The impact on farmers’ economic security varied.

**Conclusions:**

For further landscape-level irrigation developments, we recommend integrating preserving seminatural habitats and the structural diversity of the agricultural landscape in planning processes. We also recommend a shift towards more water efficient crops, evapotranspiration management, and a new funding scheme for farmers to offset rising electricity costs.

**Supplementary Information:**

The online version contains supplementary material available at 10.1007/s10980-024-01977-y.

## Introduction

Irrigation is crucial for agricultural production in many parts of the world (Velasco-Muñoz et al. 2019). However, irrigated agriculture has been found to damage ecosystems and contribute to water scarcity (Scherer & Pfister 2016), threatening human well-being (Wada et al. 2014). Concurrently, the need for irrigation is increasing due to climate change-induced alterations in rainfall patterns (Elliott et al. 2014; Fader et al. 2016; FAO 2020) and rising demands for agricultural products (Tilman et al. 2011; Davis et al. 2016). The intensification of agricultural production processes to maximize yields is a major driver of environmental degradation in Europe (Diaz et al. 2019; Pe’er et al. 2020) and has been found to threaten landscape sustainability (Plieninger et al. 2016). Especially rapid landscape changes can negatively impact biodiversity and human well-being (Tscharntke et al. 2005; Wu 2013). To preserve ecosystem services while augmenting agricultural production, pathways towards greater sustainability are needed (Rasmussen et al. 2018; Kleijn et al. 2019; Watson et al. 2021).

The concept of sustainable agricultural intensification (SI) has been suggested and advocated by both scientists and policy makers (Pretty 1997; Helfenstein et al. 2020) to accommodate the necessities of future food security while sustaining the environment and improving quality of life. SI was also the focus of the recently finished international project Sustainable Agricultural Intensification Pathways in Europe (SIPATH) carried out by the Swiss Federal Institute for Forest, Snow and Landscape Research WSL (Switzerland), Agroscope (Switzerland), and the Vrije Universiteit Amsterdam (The Netherlands) (e.g., Helfenstein et al. 2020, 2024; Debonne et al. 2022; Williams et al. 2023; Mohr et al. 2023). One research strand of SIPATH was based on a series of case studies in landscapes of distinct characteristics across Europe (see Mohr et al. 2023 and Diogo et al. 2023 for the selection procedure). One of the case study sites was located in the municipality of Santa María del Páramo, which belongs to the autonomous community of Castilla y León in northern Spain. Oral history interviews (OHIs) with elderly farmers conducted at this site in 2021 identified changes in agricultural irrigation practices and land consolidation as important factors in farm development (Mohr et al. 2023). The greater area of the study site underwent a transition to intensive agriculture in the 1960s, when the construction of reservoirs in the mountains of Castilla y León facilitated the use of irrigation water in the plains on a larger scale than previously possible with the traditional irrigation system using wells (Franco Pellitero 1986; García Martínez 2020). Spain’s full inscription in European regulations in 1986 amplified the increasingly productivist orientation of irrigated agriculture in Castilla y León (Alario Trigueros et al. 2016). From the mid-2000s onwards, sector-wise installations of new irrigation infrastructure took place within the case study region, as García Martínez (2020) and Rodríguez (2011) described. Spatially, these authors focused on the regional scale, while detailed information on the farm-scale development–including the farmers’ perspectives – remains missing. Analyzing land-use-change processes at multiple institutional and spatial scales makes it possible to capture and integrate the diverse perceptions of stakeholders (Diogo et al. 2022).

The study of so-called driving forces has evolved as a conceptual framework to understand how and why land use and landscapes change (Bürgi et al. 2005, 2022; Plieninger et al. 2016). Driving forces are regarded as the forces that cause the observed changes (Bürgi et al. 2005), and in this study, the term *process* is adopted for the entity upon which driving forces act (Bürgi et al. 2017). For the study of driving forces, an assessment over time is of value, while spatially comparative approaches can also help to understand the causes of changes (Bürgi & Russell 2001). In Spain, land use has changed dramatically over the past decades (Peña et al. 2007; Corbello-Rico et al. 2015). Like many Mediterranean countries, parts of Spain are facing land abandonment (Lasanta et al. 2017), while other parts are experiencing agricultural intensification (Serrano et al. 2024; Helfenstein et al. 2024). Shifts from traditional to intensive agriculture and the associated production growth in southern and eastern Spain were for example driven by technological innovation (Serrano et al. 2024) and agricultural policies (Peña et al. 2007).

Land-use-change processes and the impacts of change are mainly studied separately, although linking the processes and their impacts would be beneficial (van Vliet et al. 2016). In the Duero river basin, to which the study site belongs, landscape impacts of irrigation have been identified by Gómez-Limón & Riesgo (2009) and Rodríguez (2011). These authors observed a decrease in crop diversity with the reduced use of the traditional flood irrigation. The decrease in crop diversity tends to homogenize the landscape (Gómez-Limón & Riesgo 2009; Rodríguez 2011). The shift towards monoculture has been found to have contributed to the decrease in the number of farms, to have created more economic dependency of the study site region, to have left farmers with little bargaining power in the market, and to have increased contamination by pesticides (González de Molina 2001; García Martínez 2020). However, new irrigation possibilities have also been found to lead to more harvest security (Rodríguez 2011), and the replacement of flood irrigation has been associated with improved water efficiency (Fernández García et al. 2013; Carrillo Cobo et al. 2014; Eldeiry et al. 2016). Ecological, economic, and social transformations related to irrigation in the study site region are hence encountered in literature, but information is scarce on the sustainability outcomes specifically related to the most recent irrigation developments.

To address the research gaps mentioned above, we adopt a landscape-level perspective to analyze recent developments in the study site of Santa María del Páramo, where a preliminary analysis recognized the development of a large-scale irrigation system (Mohr et al. 2023). For contextualization and comparison, we incorporated a second site from the same region, with similar topographic, climatic, and socioeconomic conditions, into the study design, but for which we assumed a different irrigation development based on preliminary local information. The aim of this study is to analyze the recent development of agricultural irrigation at the two study sites as well as to study responsible multi-scale driving forces. We further intend to show related landscape-level impacts and sustainability outcomes, including farmers’ perspectives. More specifically, we ask the following research questions:

1. How did agricultural irrigation develop at two study sites in Castilla y León (Spain)?
2. What are the driving forces of the development of a large-scale irrigation system?
3. How did the development of a large-scale irrigation system impact the landscape?
4. Which sustainability outcomes are associated with the development of a large-scale irrigation system?

To address the first research question, we considered the irrigation development trajectories since 1970 and the involved institutional organization. For the subsequent analysis of driving forces, landscape change, and sustainability outcomes, we focused on recent developments of the last 20 years. We conclude the article with suggestions for enhancing the sustainability of future irrigation development in the study area and in similar contexts.

## Study sites

**Fig. 1:**
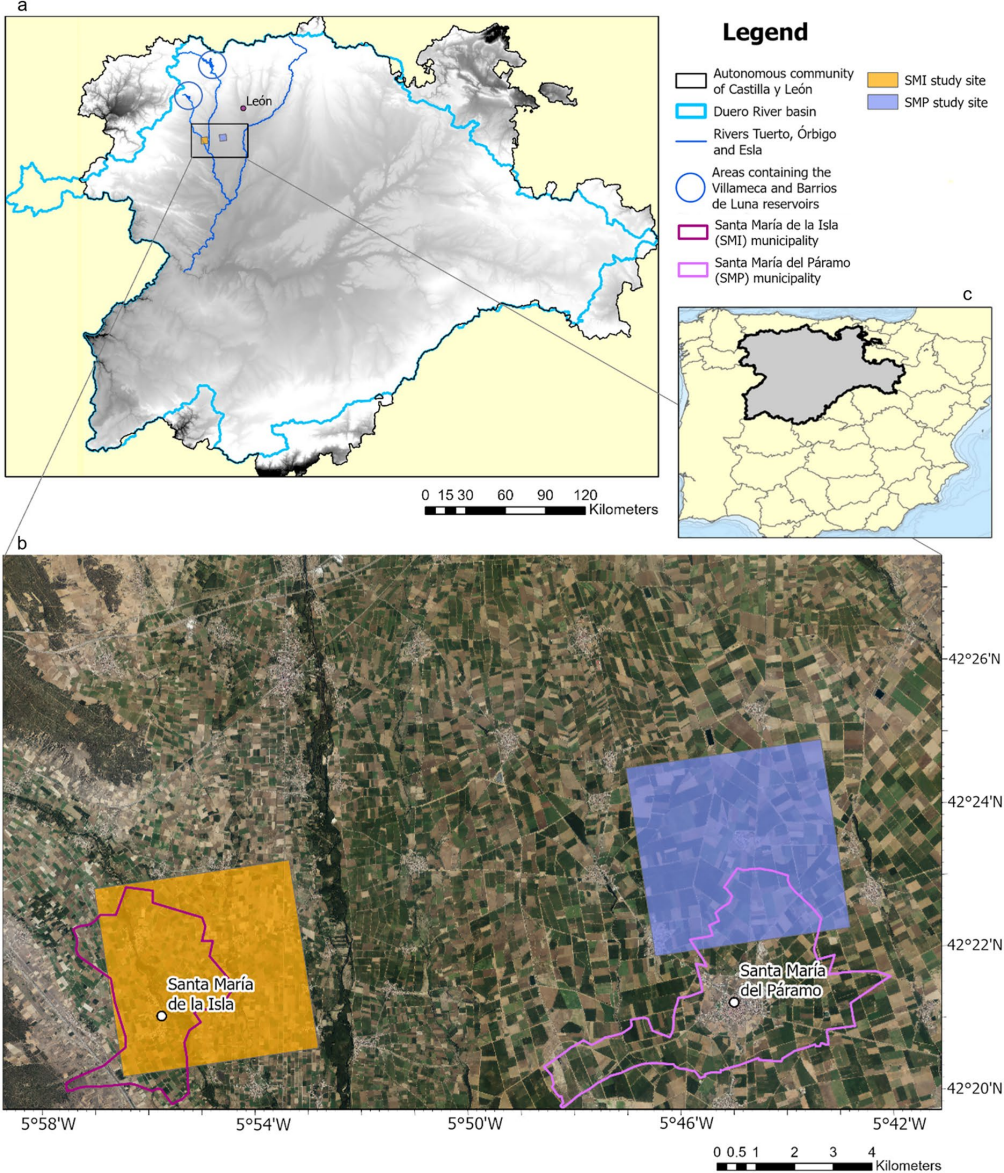
Locations of the Santa María de la Isla (SMI) and Santa María del Páramo (SMP) study sites. Map **a** shows the location of the study area in the Duero River basin and the autonomous community of Castilla y León. Map **b** shows the locations of the study sites and municipalities. Map **c** shows the location of the autonomous community of Castilla y León in Spain. Data sources: Orthophoto from Instituto Geográfico Nacional de España (IGN), administrative boundaries and hydrographic information from Confederación Hidrográfica del Duero (CHD)

We selected two study sites of 25 km^2^ with similar topography, climatic conditions, and land use (Fig. 1). One site forms part of the Santa María del Páramo (SMP) municipality, while the second forms part of the Santa María de la Isla (SMI) municipality. Both study sites are in the autonomous community of Castilla y León in northern Spain, which is governed by the *Junta de Castilla y León* regional government (Junta de Castilla y León, n.d.a). Both study sites are part of the Duero River basin (Gómez-Limón & Riesgo 2009). The basin covers 98,073 km^2^, 78,859 km^2^ of which belongs to Spain while the rest of the area is in Portugal (Pardo-Loaiza et al. 2021). The whole basin contains 75 artificial large-scale reservoirs with a total capacity to store 7500 million m^3^ of water (Pardo-Loaiza et al. 2021).

The Esla River passes to the east of the SMP study site, and the Órbigo River flows to the west of the site (Fig. 1). To the south, the fluvial terraces of the union of the Esla River and Órbigo River begin (García Martínez 2020). The Tuerto River passes through the SMI site. The two study sites have a smooth topographic relief and similar climatic conditions. A Mediterranean-type climate with continental characteristics, such as low rainfall and large differences in summer and winter temperatures, is prevalent (Gómez-Limón & Riesgo 2009). The average annual temperature in the region is 11.9 °C, and the annual rainfall is around 460 mm (Junta de Castilla y León, n.d.b). Irrigated agriculture is of great economic importance at both study sites (Ayuntamiento de Santa María de la Isla, n.d.; García Martínez 2020).

## Material and methods

The combination of quantitative and qualitative data leads to a comprehensive understanding of land-use and landscape change (Cheong et al. 2012; Kleemann et al. 2017) and enables the analysis of multiple sustainability dimensions in the context of irrigated agriculture (Antunes et al. 2017). We therefore used a mixed-methods approach based on qualitative data from interviews and document analysis, and quantitative data from aerial photograph analysis. After gathering and analyzing the data as presented in the following sections, we followed the procedure of combining the quantitative and qualitative data as described by Plano Clark & Ivankova (2016).

## Stakeholder interviews

We conducted eighteen semi-structured interviews at the study sites in August and September 2022. The interviews served as the primary data source for studying the irrigation development and the large-scale irrigation system’s driving forces and sustainability outcomes. We interviewed ten farmers, as they are the most direct users of the irrigation system, making use of contacts established during the SIPATH project as a starting point. To account for the historical dimension of the research questions, we preferred older farmers as interviewees, with the farmers’ ages ranging from 46 to 70. We found further interviewees through snowball sampling (Flick 2016). To expand the variety of stakeholder perspectives, we conducted interviews with members of irrigation communities, an irrigation union, and the regional government. These interviewees provided detailed information on economic and social sustainability outcomes. To gather further information on environmental sustainability outcomes and to obtain a more differentiated view, we interviewed three environmental experts (following Kienast & Helfenstein 2016). All interviewees were selected based on their professional role/function. This resulted in all interviewees being male, except in one case where a couple was interviewed who jointly managed their farm. We tailored the interview questions to the expertise of the different stakeholder groups and we conducted all interviews in Spanish, together with a local project partner. We transcribed the interviews with the software MAXQDA (VERBI, Berlin, Germany) and subsequently translated them to English for further analysis.

As proposed by Mayring (2016), we segmented the transcripts into codes and subcodes, to compile the results in written, tabular, and graphic form. We coded for driving forces, landscape changes, and sustainability outcomes. While we coded the landscape changes inductively, we coded the driving forces deductively based on the driving forces concept (Bürgi et al. 2005, 2022). The deductive codes covered political and institutional; economic; technological; cultural and personal; and natural and spatial driving forces. For the coded driving forces, we inductively created subcodes to better capture the relevant topics mentioned. We coded not only for driving forces of change, but also for driving forces impeding change. For the sustainability assessment, we based the codes on the three main dimensions of sustainability as distinguished in the Brundtland report (Brundtland 1987) and subcoded the dimensions inductively, based on the perceived sustainability outcomes of interviewees. Quantitative data from booklets provided by irrigation communities complemented the interview data (see below).

## Document analysis

To complement the information provided by the interviews, we further conducted a document analysis. We based the procedure for the document analysis on Mayring (2016) and Flick (2016). The sample of documents consisted of material referred to or provided by the interviewees that were directly related to the irrigation development and sustainability outcomes at the study sites. For example, stakeholder websites or booklets produced by irrigation communities were included as document types. The information from these documents was added to the corresponding interview coding categories and provided contextual evidence as well as numbers to support the interview statements.

## Aerial photograph analysis

For the landscape assessment, we included land cover and indicators of landscape structure, due to the importance of structural landscape elements for non-market outcomes (Helfenstein et al. 2020). The landscape structure indicators covered the number of field trees, the length of hedgerows and tree lines, and the field size (Helfenstein et al. 2024).

We used orthophotos from the *Instituto Geográfico Nacional de España* (IGN) for the landscape assessment because the spatial resolution of satellite images is often too coarse for determining structural characteristics at the agricultural landscape scale (Persson et al. 2010; Morgan et al. 2017). The two time points selected were the years 2002 and 2017, to include the change to the large-scale irrigation system in SMP. The orthophoto from the year 2002 stemmed from a flight between June and August with a spatial resolution of 0.5 m. The orthophoto taken in July 2017 had a spatial resolution of 0.25 m. We classified land-use and landscape elements through visual interpretation. The software ArcGIS Pro (version 2.9.5; Esri, Redlands, CA, USA) served to carry out the landscape mapping. We first created a non-agricultural land mask and mapped parcels, using feature lines. We then marked land-use with feature points based on the European Nature Information System (EUNIS) habitat classification (EEA 2019), which we adapted according to Helfenstein et al. (2024). We transformed feature lines and feature points to land-use polygons, and mapped landscape elements such as tree lines, hedgerows, and field trees with feature lines and feature points, based on Helfenstein et al. (2024). We calculated statistics and created maps with ArcGIS Pro for visual representation of the results.

## Results

### Irrigation development

During the period analyzed (1970 to 2022), the main water source at both study sites was surface water, provided by reservoirs. Farms in SMP are supplied by the *Barrios de Luna* reservoir, which began to operate in 1956 and has a water capacity of 300 million m^3^ (Confederación Hidrográfica del Duero 2019b). At the SMI site, farms located on the left bank of the Tuerto River are supplied by the *Barrios de Luna* reservoir, while farms on the right bank are supplied by the *Villameca* reservoir. The latter was commissioned in 1947 and can store up to 20 million m^3^ of water (Confederación Hidrográfica del Duero 2019c). From the reservoirs (see Fig. 1 for their locations), the water is transported downstream through rivers and channels.

**Fig. 2:**
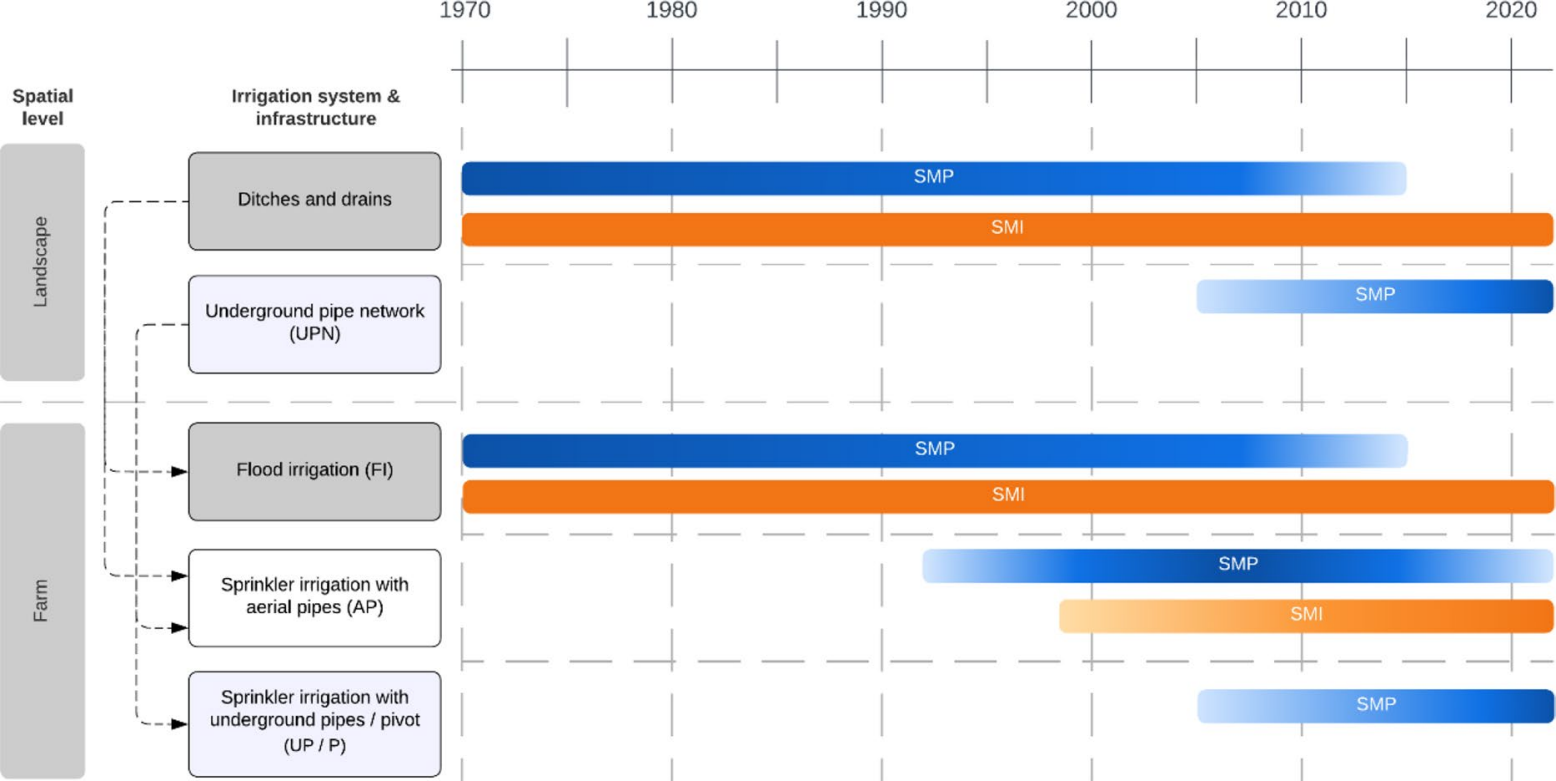
Development of irrigation systems and infrastructure used to distribute the reservoir water on the landscape and farm level at the Santa María del Páramo (SMP) and Santa María de la Isla (SMI) study sites since 1970. Affiliations between the landscape and farm levels are displayed with dotted arrows. Color gradients indicate shifts in the use of irrigation systems and infrastructure at the study sites

From 1970 onwards, all interviewees had their own farms and irrigated them by flood (Fig. 2). The water was transported through the landscape via ditches to the farms. Surplus water that had collected in drains after irrigation could also be used by farmers whose lands were adjacent to drainage ditches. For on-farm distribution, farmers created furrows on their plots where the water from irrigation ditches would flow through when the flood gate was opened. With great physical effort, farmers would direct the water through the furrows and periodically divert it to different sectors. Farmers in SMP completely stopped irrigating by flood between 2010 and 2017 (Fig. 2). In SMI, the farmers were still irrigating partly by flood at the time of the interviews.

**Fig. 3:**
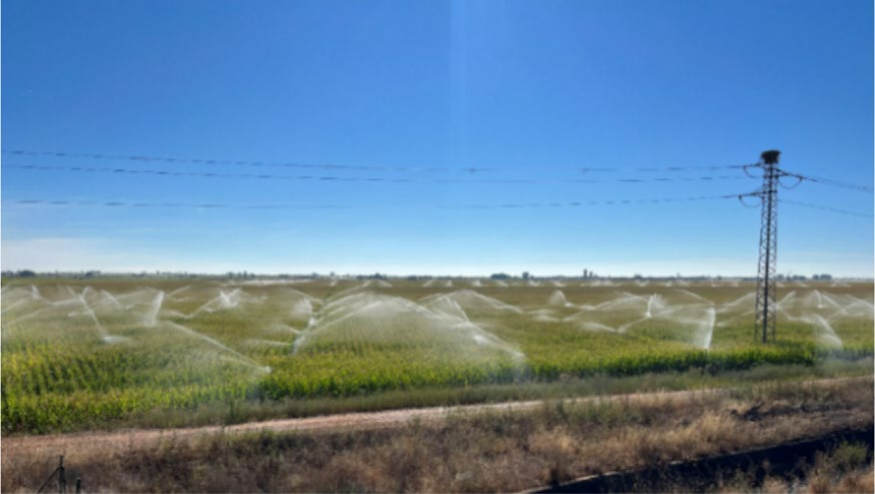
On-farm sprinkler irrigation in the Santa María de la Isla (SMI) study site in 2022 (photo taken by Fabienne Frey)

Aerial pipes were partially introduced on farms at the study sites in the 1990s, but the water distribution through the landscape still relied on the network of ditches. For the aerial piped irrigation, diesel-fueled motor pumps or engines would take water out of an irrigation or drainage ditch and pump the water through the attached pipes, which were laid on the farm in a grid. Sprinklers were connected to the aerial pipes and their heads would distribute the water on the plots (Fig. 3). After every irrigation campaign, farmers would remove the aerial pipes from their plots for harvesting. Overall, the ratio of land irrigated by aerial pipes to flood-irrigated land ranged from 10 to 50% per farmer. Aerial pipes continue to be used at both study sites, but the ratio has decreased at the SMP site since the establishment of the large-scale irrigation system (Fig. 2).

Colloquially, interviewees used the term *modernization* to refer to the large-scale underground pipe network (UPN). The establishment of such a large-scale network solely concerned the SMP site at the time of the interviews. This study site forms part of the *Comunidad General de Regantes del Canal del Páramo*, where installations started in 2005 and continued to be executed sector-wise in the following years.

**Fig. 4:**
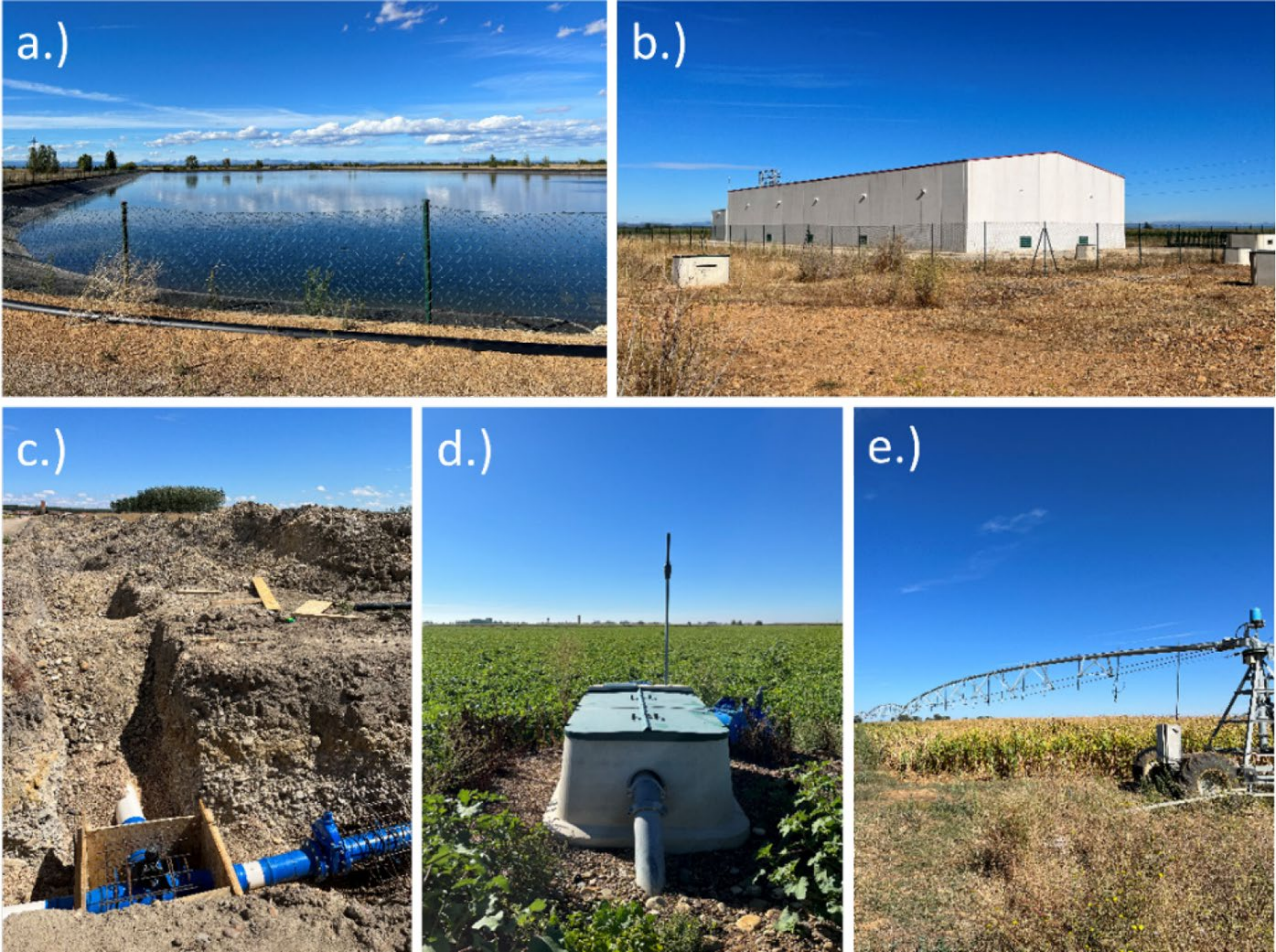
Underground pipe network (UPN) irrigation infrastructure in the Santa María del Páramo (SMP) study site: raft (**a**), pumping station (**b**), underground pipes being installed (**c**), hydrant (**d**), and pivot (**e**) (photos taken by Fabienne Frey in 2022)

With this UPN irrigation system, the reservoir water is transported to rafts through the previous channels, but additional rafts are also constructed (Fig. 4a); from the rafts, the water is redirected to pumping stations (Fig. 4b) through underground pipes. At the pumping stations, the water is pressurized using electricity. A network of underground pipes then transports the water to hydrants on farms (Fig. 4c, d), replacing the previous network of ditches. A farmer can choose to install on-farm underground pipes, which are connected to sprinklers that distribute the water on the field. Irrigating with underground pipes can be controlled remotely on demand with a smartphone application. According to farmer statements, the same sprinklers as with aerial pipes can be used (Fig. 3). It is also possible to connect the removable aerial pipes to the hydrant or to irrigate with a pivot (Fig. 4e).

As an obligatory precondition for the UPN, land consolidations are carried out. Parcel size is increased, properties are joined, small paths are eliminated, roads are widened, ditches are eliminated, and the underground pipe infrastructure is installed. The land consolidation with UPN installation requires approval by landowners in a general assembly of the according irrigation community. Voting began in 2000 in the case study region. While the project was approved in SMP in the second round of voting, multiple rounds of voting turned out negative in SMI and a positive outcome was only achieved shortly before the interviews.

## Institutional organization

**Fig. 5:**
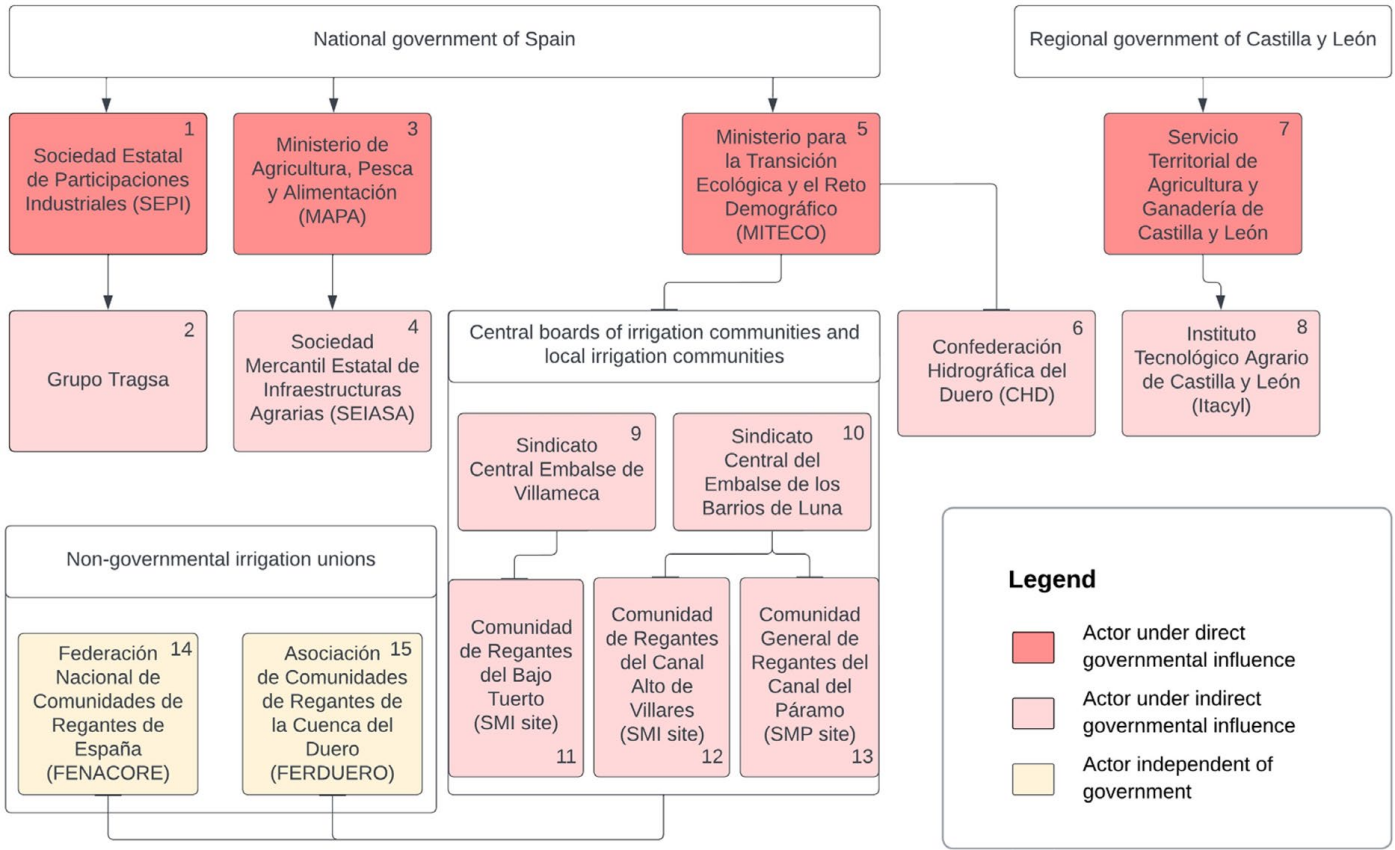
Overview of institutional actors in the Santa María del Páramo (SMP) and Santa María de la Isla (SMI) study sites along with the data-based estimation of governmental influence. Linkages ending with arrows represent the establishment of an entity by an actor, and linkages without an arrow indicate affiliations between actors. Actors are listed with their Spanish names and are numbered to follow the elaborations in the text, where the numbers appear in brackets

The stakeholders involved in the irrigation development studied here included individuals, such as farm-level actors or non-agricultural landowners, and institutional actors, such as irrigation companies. With the UPN, institutional actors, including both governmental and non-governmental entities, were deeply involved. We found the linkages between institutional actors to be complex, and we found differences in the type of governmental influence on institutional actors (Fig. 5).

On the national level, the *Ministerio de Agricultura, Pesca y Alimentación* (*MAPA,* 3) is the department within the general state administration that is responsible for agriculture, fisheries, and food (Ministerio de Agricultura, Pesca y Alimentación, 2020). As an instrumental company of the *MAPA,* the *Sociedad Mercantil Estatal de Infraestructuras Agrarias* (*SEIASA*, 4) was established in 1999 (Ministerio de Agricultura, Pesca y Alimentación, n.d.) to promote and contract investments in irrigation development and consolidation works (Sociedad Estatal de Infraestructuras Agrarias 2013). A further state company involved in the execution of irrigation projects is *Grupo Tragsa* (2) (Grupo Tragsa, n.d.), which forms part of the *Sociedad Estatal de Participaciones Industriales* (*SEPI*, 1) public law entity. Under the *Ministerio para la Transición Ecológica y el Reto Demográfico* (*MITECO*, 5) state department, the *Confederación Hidrográfica del Duero* (*CHD*, 6) manages the water of the Spanish part of the Duero River basin (Confederación Hidrográfica del Duero 2019a). The confederation is responsible for the administration and control of the public water domain, including the release of water from reservoirs to irrigation communities (Confederación Hidrográfica del Duero 2019a).

On the regional level, the *Servicio Territorial de Agricultura*, *Ganadería y Desarrollo Rural de León* (7) is the governmental department of Castilla y León concerned with agriculture, livestock, and rural development (Agricultura y Ganadería, n.d.). Attached to this ministry, the *Instituto Tecnológico Agrario de Castilla y León* (*ITACyL*, 8) was created to promote the development of Castilla y León’s agri-food industry and agricultural innovation, including agricultural irrigation (Itacyl, n.d.).

Local irrigation communities are corporations under public law, created by ministerial orders. Their purpose is to manage a water resource and distribute it to its members. A farmer automatically becomes a community member by irrigating his or her land. The leadership positions in an irrigation community are mostly occupied by active or former farmers. The *Comunidad General de Regantes del Canal del Páramo* (13), of which the SMP farmers are part, is responsible for a total of 17,000 irrigated hectares of farmland across 29 villages (Ayuntamiento Santa María del Páramo, n.d.b). The irrigated area that belongs to the municipality of SMI is divided into two communities. The communities on the right bank of the Tuerto River belong to the *Comunidad de Regantes del Bajo Tuerto* (11)*,* which was created in 2021 after a fusion of four smaller communities and covers a total of 1,867 irrigated hectares (Comunidad de Regantes del Bajo Tuerto 2021). On the left bank of the Tuerto River, the irrigated area of the municipality of SMI is part of the *Comunidad de Regantes del Canal Alto de Villares* (12)*,* which encompasses a total of 2254 hectares (Comunidad de Regantes del Canal Alto de Villares 2019).

Central boards of reservoir water users include all the irrigation communities supplied by a reservoir and handle administrative tasks. The local irrigation communities of the study sites are part of the *Sindicato Central del Embalse de los Barrios de Luna* (10) and the *Sindicato Central Embalse de Villameca* (9), which are dependent on the state department *Ministerio para la Transición Ecológica y el Reto Demográfico* (*MITECO*, 5). Associations independent of state bodies have also been created to defend the interests of irrigators. On the national level, the *Federación Nacional de Comunidades de Regantes de España* (*FENACORE*, 14) brings together irrigation entities (Federación Nacional de Comunidades de Regantes de España, 2023). On the regional level, the *Asociación de Comunidades de Regantes de la Cuenca del Duero* (*FERDUERO*, 15) is a non-governmental association of 134 irrigation communities of the Duero River basin (Asociación de Comunidades de Regantes de la Cuenca del Duero 2019).

## Driving forces

Overall, governmental entities and irrigation unions were more involved with the UPN than with on-farm infrastructure, while driving forces for on-farm infrastructure were mainly related to farmers. The driving forces are displayed in Table 1, and the most relevant driving forces are described below.

**Table 1:** Overview of categorized perceived driving forces of the underground pipe network (UPN) establishment and associated on-farm infrastructure, differentiated into four spatial scales. Driving forces found for the UPN and on-farm infrastructure are listed first and appear in bold. Driving forces solely prevalent with on-farm irrigation infrastructure are indicated with (F). The rest of the driving forces account for the UPN alone

	National (Spain) and international	Regional (Castilla y León)	Landscape (study sites)		Individual / farm level
			SMI	SMP	
Political and institutional driving forces	– Agricultural policies – Environmental activism – International contracts – Financial incentives	– Agricultural policies – Rural development – Transformation of the agricultural sector – Financial incentives – Information campaigns	– Irrigation community structure – Merging of irrigation communities – Personal disputes – Individual action – Pandemic – Land ownership	– Irrigation community structure – Land ownership	– **Land ownership**
Economic driving forces		– Water use efficiency – Crop diversification – Market growth and commercialization – Interests of agro-industrial companies	– Irrigation costs – Interests of agro-industrial companies	– Irrigation costs – Farm size (F)	– **Water use** **efficiency** – **Productivity** – **Optimization of crop conditions** – **Irrigation costs** – Land value – Crop diversification – Investment rentability – Farm size – Parcel size
Technological driving forces				– Irrigation infrastructure age	– Irrigation system characteristics (F) – Irrigation infrastructure availability (F)
Cultural and personal driving forces			– Population age structure – Neighborhood effect	– Population age structure – Irrigation history – Neighborhood effect	– **Work** **comfort and quality of life** – **Interprofessional exchange** – Motivation to move forward – Motivation to save resources – Skepticism towards the new – Mentality – Decrease in family help (F)
Natural and spatial driving forces	– Climate change	– Droughts	– Water availability – Extent of irrigated area	– Farm location – Extent of irrigated area	

On the political and institutional side, governmental water management policies to reduce water losses were evident. To reduce water losses, the UPN was mentioned as being indispensable because it is supposed to save up to 30% of water compared with flood irrigation. Farmers noted the governmental water management ambitions: “Well, the rules, what they tell us is that (…) what we have always been told is that we must save water. That water must be optimized (…) optimize and save as much as possible […]” (SMP05). Fostering rural development, preventing emigration from the countryside, and transforming the agricultural sector in Castilla y León towards more horticultural production are further goals of the regional agricultural department, with the UPN viewed as indispensable: “without modernization (…) it does not go anywhere” (IU01). At both study sites, the regional government collaborated with irrigation communities and irrigation companies to convince farmers of the benefit of the UPN. Informative talks were held in villages because “[…] the modernization process requires good communication with farmers. There is a part that is psychological, because you have to convince them […]” (IC01). The Spanish national government, the regional government of Castilla y León, irrigation companies, and banks also started to provide funds, loans, and special tariffs for farmers to foster UPN establishment. An institutional driving force found to impede UPN development was land ownership. Farmers do not own all the agricultural land they work on, but all landowners were able to vote on the project, including non-agricultural landowners. Non-agricultural landowners tended to reject the UPN at both study sites and to delay a positive vote in the communities. They were often unwilling to invest in irrigation infrastructure because they, for example, lived outside the region or did not have a farming background, and did not see the necessity for change. Land ownership was found to influence not only the large-scale network but also the on-farm irrigation infrastructure. Once underground pipes had been installed up to the farms, as in the case of SMP, some landowners still did not install on-farm underground pipes. In these cases, farmers needed to continue to irrigate their leased farms with aerial pipes.

Economically, a frequently mentioned reason to favor the UPN was increased water use efficiency. With the remotely controlled system, irrigation can be adjusted to the crops’ water needs better than with the fixed flood irrigation cycle. A UPN means that water is available when a crop needs it, which can reduce the water stress of a crop. In the same vein, the connection of farms to the UPN leads to increased productivity: “[…] what is clear (…) is the increases in production with modernization, nobody doubts that” (IU01). Another economic driving force that was recognized is the possibility of increasing farm size, as the UPN facilitates the irrigation of a larger area. Farmers mentioned the joining of properties in the scope of the land consolidation as a further reason to approve the UPN. A fragmentation of farms is associated with higher production costs than larger parcels joined at the same location. This rationale was also found within the regional government: “We do not modernize if it is not concentrated. Why? Well, because what we want is farms that are as viable as possible, in surface, having the lowest number of farms possible, of the largest area, trying to concentrate by farms […]” (RG01). However, farmers at both study sites rejected the UPN establishment at first because of its high costs and doubts about investment rentability, and irrigation costs continued to influence the project realization after the approvals. The high costs of the UPN establishment led to sector-wise project realization in SMP and delays of project realizations in SMI. Eight of ten farmers mentioned irrigation costs influencing their on-farm infrastructure decisions. For example, the cost of a pivot was cited as having prevented some farmers in SMP from buying one.

Technological driving forces were the least frequently mentioned category. However, the infrastructure age was influential in the irrigation community of SMP. UPN installations began where flood irrigation infrastructure was the oldest. Farmers who were then connected to the UPN preferred to have the new technology of on-farm underground pipes operated by phone rather than manually connecting their aerial pipes to the hydrant.

Culturally, a conservative mindset was said to have contributed to the resistance towards the UPN. Farmers, especially elderly ones, said that they had been skeptical of the new system. The doubts concerned the unknown and the rentability of the new system. With a generational shift in agriculture in SMP, a change in mentality started. The younger farmers were said to be more willing to take risks and invest, while farmers close to retirement were less willing to invest. Farmers also overcame their doubts regarding the investment amortization by talking to irrigators who already had the network established. The exchange with other farmers further helped interviewees with decisions about the on-farm irrigation infrastructure, such as the installation of a pivot or underground pipes after the UPN establishment. The most frequently mentioned driving force in the cultural and personal driving forces category was work comfort and quality of life. Farmers approved of the UPN and introduced underground pipes or pivots on their farms due to the high labor intensity and associated mental stress of flood irrigation. Flood irrigation requires physical effort and is costly in time, even requiring irrigation during night-time.

Regarding natural driving forces, droughts accelerated the approval of the UPN at both study sites. While the farmers were not convinced of the necessity of the UPN beforehand, the increasing water scarcity associated with climate change enhanced their willingness to change the irrigation type. The spatial driving force of farm location was prevalent because when irrigating by flood, farmers downstream are disadvantaged compared with those upstream. Farmers downstream have already approved the new system, while those located upstream still rejected it.

The temporal difference in the UPN approval between the two study sites was attributed to the irrigation history of the SMP community. Farmers in SMI had already been irrigating with the river, while Páramo was one of the poorest regions in the province of León until the water from the *Barrios de Luna* reservoir became available in 1956. Subsequently, a shift from a subsistence economy to larger-scale production of irrigated crops started. Farmers in SMP experienced the benefits of irrigation changes and, therefore, approved the new system earlier. Furthermore, young farmers were successively incorporated into agriculture in SMP, but young farmers leaving SMI resulted in an older population age structure and more resistance towards the UPN, delaying the project realization in SMI. More resistance in SMI was also attributed to the land ownership configuration. At the SMI site, poplar plantations are prevalent, and owners of poplar plantations constantly voted against the UPN because poplars do not need to be irrigated.

While both study sites are the same size, their irrigated area is managed by different irrigation communities, which differ in the total extent of the irrigated area for which they are responsible. In SMI, the irrigation communities encountered difficulties in obtaining political support, due to the small amount of irrigated area. In SMP, the irrigated area is managed by one community, and its large spatial extent was mentioned to have facilitated the establishment of the UPN. The structure of irrigation communities was also said to play a role in the UPN establishment, as the large size and number of personal resources of the SMP community facilitated the UPN establishment. In contrast, the smaller irrigation communities in SMI lacked personnel. The irrigation communities of SMI were merged in 2021, contributing to the final UPN approval.

## Landscape changes

Cropland was the dominant mapped land-use type in both study sites at both of the analyzed time points, comprising 88% of the SMP site and 81% of the SMI site in 2017 (for absolute values, see Supplementary Information). Every farmer interviewed had irrigated all crops since the start of their agricultural activity. At the time of the interviews, corn was planted in up to 70% of a farmer’s cultivated area in SMP, while the cultivation of beans, beetroot, and wheat had decreased since farmers started working in agriculture, or even completely stopped in some cases. In SMI, corn became popular, but potato also became an important crop. Wheat, sunflower, barley, and beetroot were cultivated only to a minor extent.

**Fig. 6:**
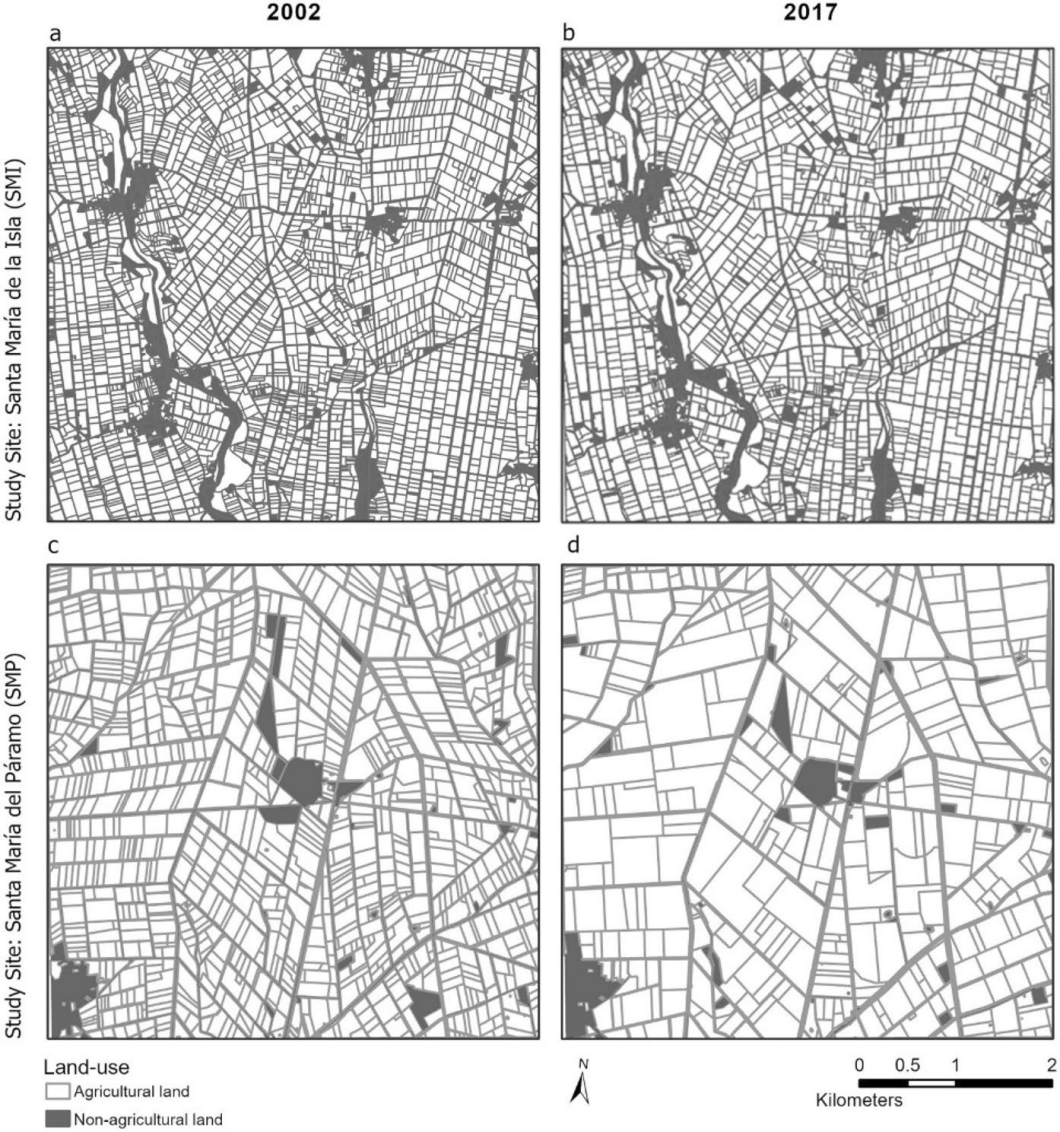
Field structure, agricultural and non-agricultural land use in the two study sites in 2002 and 2017. Each study site covers 25 km.^2^

**Fig. 7:**
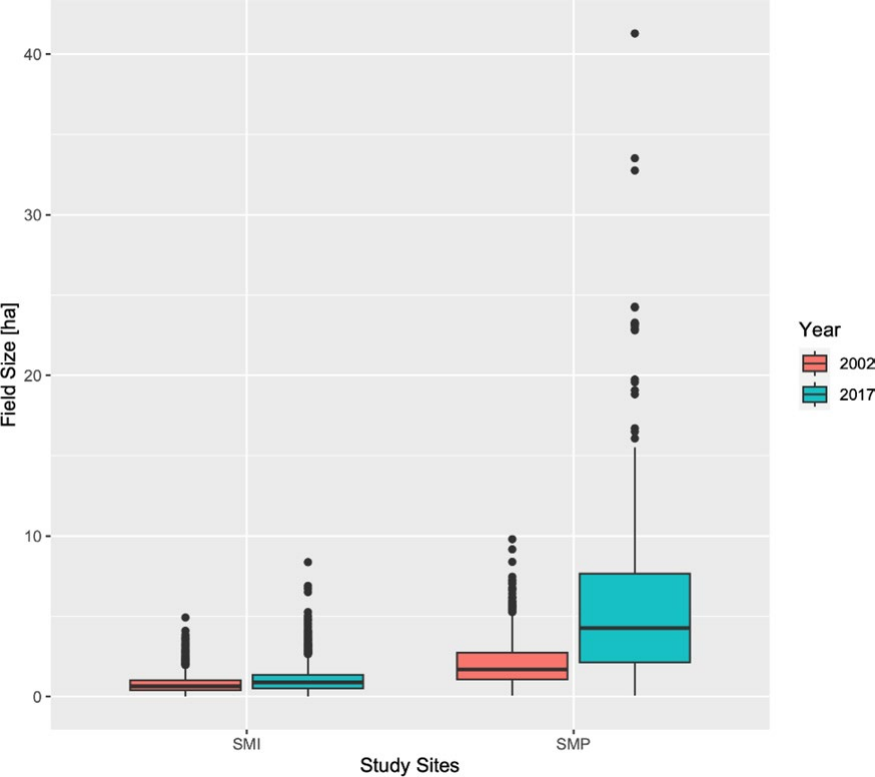
Boxplots showing medians and quartiles of the field size of crops at the Santa María de la Isla (SMI) and Santa María del Páramo (SMP) study sites in 2002 and 2017, based on aerial photograph analysis

The remaining areas of the study sites comprised other agricultural land uses, such as intensive orchards and fruit production, shrub plantations, extensive grassland, and field margin vegetation, and non-agricultural land uses, such as forest plantation, barren land, abandoned land, forest, settlements and roads, wetlands, and water (see Supplementary Information). Agricultural land dominated over non-agricultural land in both study sites (Fig. 6). The total agricultural area was larger in SMP than in SMI, both in 2002 and in 2017. It increased from 2318 to 2336 hectares, while the total agricultural area in SMI decreased, from 2154 to 2125 hectares. Crop field size increased between 2002 and 2017 at both study sites, but to a notably greater extent in SMP (Fig. 6). Whereas the median increased from 0.65 to 0.88 hectares in SMI, it rose from 1.69 to 4.27 hectares in SMP (Fig. 7). Maximum field size at the SMP study site increased from 9.8 to 41.29 hectares. Interview statements confirmed the increases in field size and attributed them to the land consolidations, which started in 2005 for the installation of the UPN in SMP.

**Fig. 8:**
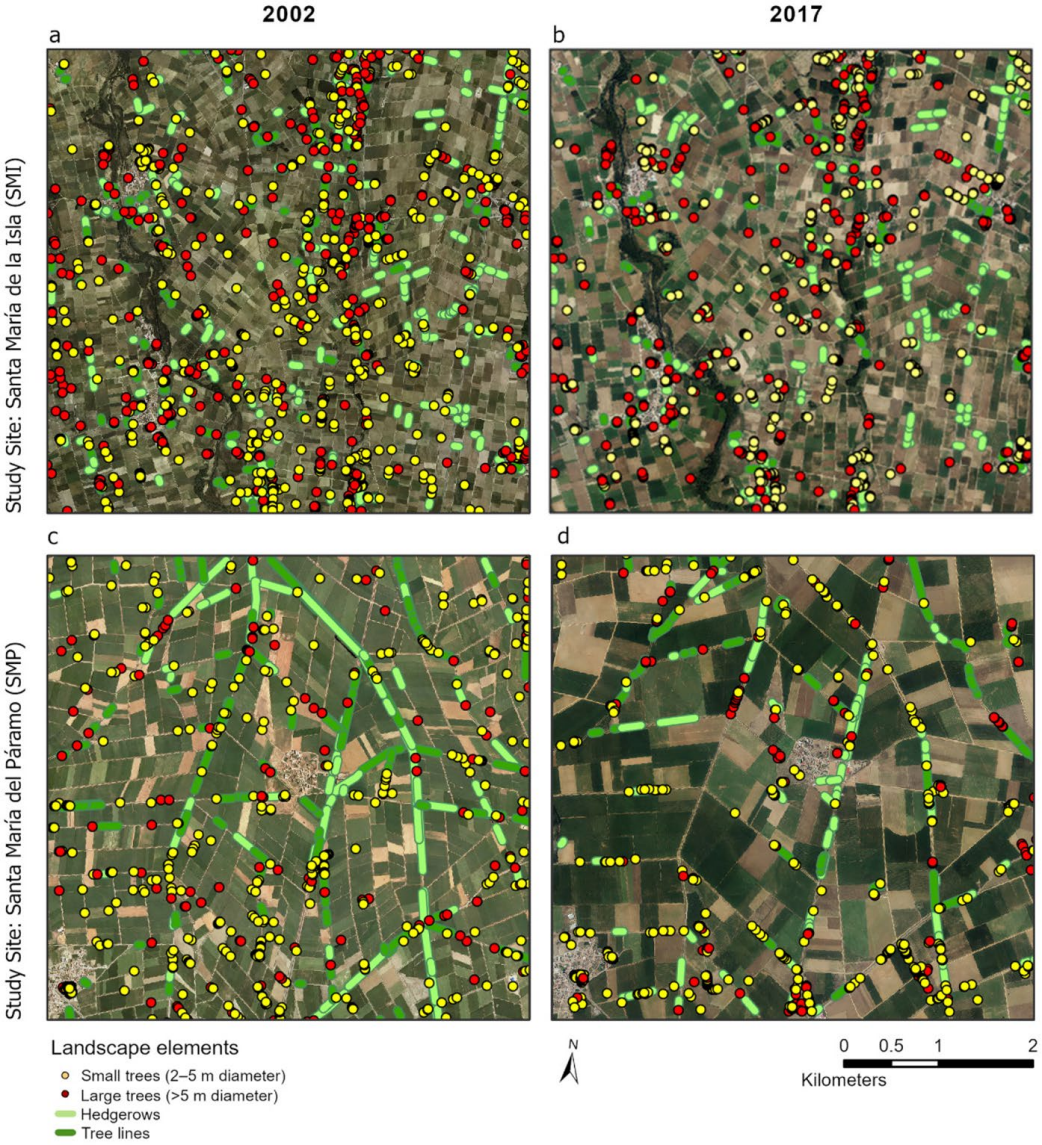
Small trees, large trees, hedgerows, and tree lines detected in the two study sites in 2002 and 2017. Each study site covers 25 km.^2^ (orthophotos provided by Instituto Geográfico Nacional de España (IGN))

**Fig. 9:**
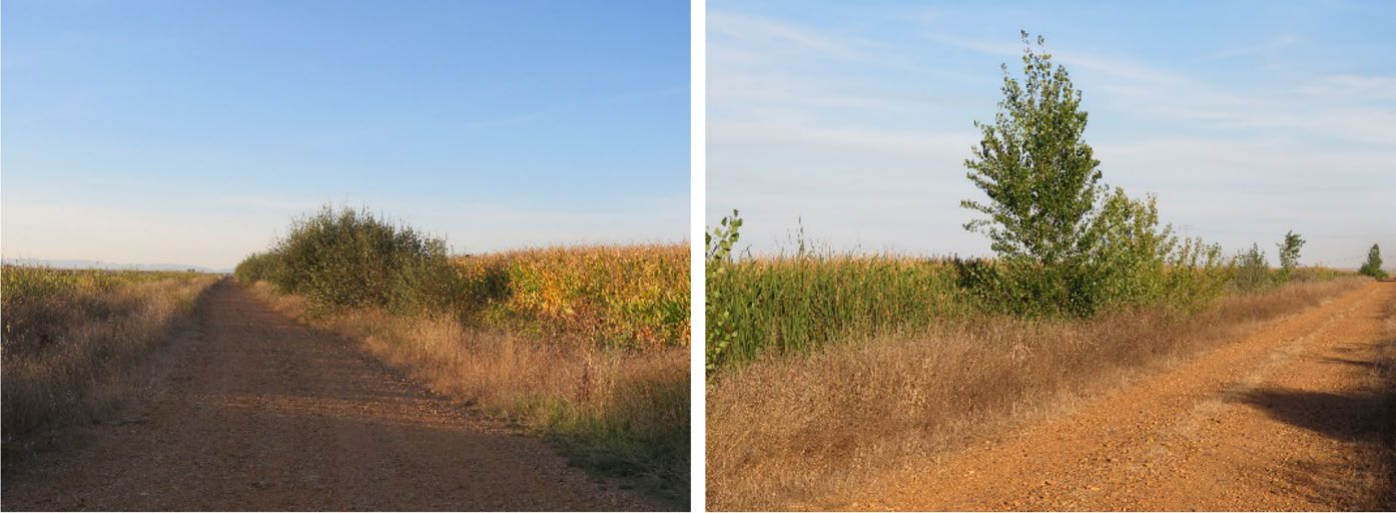
Landscape elements in the Santa María del Páramo (SMP) study site that have reduced in amount: hedgerows (left) and a sparse tree line (right) (photos taken by Virginia Ruiz‐Aragón in 2024)

From 2002 to 2017, the number of small and large trees decreased in SMI and in SMP (Fig. 8). In SMI tree counts decreased from 1,053 to 948, and in SMP from 507 to 451. Interviewees in both study sites referred to trees along roads being removed to prevent car accidents. Farmers at both study sites also removed trees on their land, as the tree roots either damaged irrigation ditches or hindered agricultural practices. The initial difference between the study sites in the number of trees could be related to field size, which was smaller in SMI already in 2002, hence allowing more individual trees between fields. While the length of hedgerows and tree lines mainly stayed the same in SMI, it decreased notably in SMP until 2017, from 17.5 to 7.7 km (hedgerows, see Fig. 9) and from 13.6 to 6.7 km (tree lines, see Fig. 9). The decrease was attributed to the land consolidation carried out to install the UPN, in the scope of which parcels were enlarged and vegetation was removed: “All the trees have also disappeared due to modernization, as the plots are large (…) to make the roads, everything was removed” (SMP01). Farmers in SMP also recounted that trees remaining along ditches had to be removed, as they started to dry out due to the absence of water in ditches after the UPN establishment.

## Sustainability outcomes

Regarding environmental sustainability outcomes, the SMP irrigation community said that the UPN led to a reduction in water consumption in the community, due to its high water use efficiency, and some farmers in SMP reported a decrease in irrigation water consumption after the installation of the UPN compared to when they used to irrigate by flood. However, the new irrigation system does not seem to have influenced annual water consumption at the regional level. Data from the *Barrios de Luna* reservoir shows variations between years without any detectable trend. As members of the irrigation union and irrigation communities explained, the annual water quantity consumed for irrigation depends on the amount of water available in a reservoir, and the variations between years are related to climatic conditions. In years of drought, the hydrographic confederation may even allow to empty the reservoir further than in other years, to secure irrigation. Environmental experts mentioned rebound effects associated with the increased water use efficiency with the UPN, for example, the irrigated area was expanded or more water-intensive crops were cultivated. Furthermore, all environmental experts related the UPN and its land consolidation to biodiversity losses because habitat loss and fragmentation occur with the increase in parcel size and the removal of structural landscape elements. The decrease in surface water with the UPN was also described as impacting flora and fauna negatively.

The social dimension was the most positively perceived sustainability dimension. Farmers noted increased occupational well-being with the UPN and described the irrigation system as “ideal” (SMP03). They reported a decrease in working hours and a change in working time and comfort, as they no longer had to irrigate during the night. Farmers without a UPN at the time of the interviews wished for one: “Well, I always tell my wife that the worst of my profession is irrigation. It’s the hardest thing, you don’t depend on yourself, you depend on other people, many things, that’s why you want them to concentrate, to modernize (…) because I see, I have friends who already have the modernization and their life is much easier. In fact, when we start the work that is in the summer, they are more relaxed” (SMI03). However, one SMP farmer stated that he felt less free in his decisions concerning sowing practices since the UPN: “They tell you, if you don’t sow this (…) you have to sow this, this and this, because you have so many liters of water. You can no longer sow what you want. You have to sow what they give you to water. There is no more. You are no longer totally free as when you watered by flood, you are limited” (SMP03). Some farmers apparently felt that the fixed amount of water allocated restricted their freedom of choice regarding the crops they could grow. Besides this, all farmers stated that the mental stress caused by irrigation disappeared with the UPN, while farmers without a UPN mentioned still experiencing irrigation-related mental stress.

In the economic dimension, production increases were prevalent with the UPN: “Productions have increased tremendously, to the point that, for example, a hectare of corn in an unmodernized area gives you 11 thousand, 12 thousand kilos per hectare. In a modernized area it reaches 16 thousand” (RG01). This corresponds to an increase by 33% to 45%, enabled through higher water and fertilizer use efficiency with the new irrigation system, as explained in the interviews. However, farmers began to incur expenses for the new irrigation infrastructure and its use, especially its electricity supply. According to some farmers, net farm income has decreased despite higher yields, mainly due to rising electricity prices. Other farmers could not estimate changes in net income, and some farmers reported an increase in income. The relationship of the new irrigation system to net farm income hence could not be determined in this study, but economic farm security seems contested.

## Discussion

## Development and driving forces of large-scale irrigation

The irrigation development reconstructed for the two study sites fits with the trend that has been recognized in other semi-arid regions, where pressurized water distribution has become increasingly common on the landscape level (Díaz et al. 2012; Fernández García et al. 2020; Sanchis-Ibor et al. 2021) and the on-farm irrigation development from flood to sprinkler irrigation has increased, especially since the 2000s (Nogués & Herrero 2003; Herrero et al. 2007; Casterad et al. 2018).

As for the driving forces of the large-scale irrigation development, political and institutional driving forces were greatest in number in this study, which fits with the findings of a meta-study on landscape and land-use change in Europe (Plieninger et al. 2016) and is in accordance with the privileged position of irrigated agriculture in Spanish policy (Lopez-Gunn et al. 2012). Despite the privileged position on the national level, this study identified land ownership as a relevant landscape-level institutional driving force slowing down UPN establishments. This finding was not encountered in the literature, probably because most other studies have been focused on driving forces that intensify processes, while in this study driving forces that impeded changes were also incorporated.

Technological driving forces were less relevant than in other studies on land-use change in Europe (Jepsen et al. 2015; van Vliet et al. 2015). This may be related to the research object, an irrigation development comprising technological changes. Jepsen et al. (2015) identified irrigation per se as a technological driving force of land-use change in Europe. The small number of technological driving forces identified here may also mean that these play a less major role in irrigation development than other driving forces. Social organization was found to be influential here and is a driving force that has been highlighted in studies on land-use / cover change as well (Lambin et al. 2003; Axinn & Ghimire 2011; van Dijk et al. 2015); the findings regarding population age structure fit with the existing research. Farmers close to retirement were found to be less willing to invest in the new irrigation system than young farmers. According to Fernández García et al. (2020), the early adopters of new technologies are usually younger farmers, and van Vliet et al. (2015) found that young farmers are more inclined to make considerable farm changes than old farmers. The latter authors explained this intergenerational difference by stating that young farmers have a longer career ahead of them for investments to become profitable. Interviews at the study sites indicated that older farmers were also generally more skeptical about the new irrigation system. While financial incentives to foster irrigation changes were encountered in the literature, the psychological aspect of convincing farmers was not encountered. This may be related to the relatively informal nature of the measures used. Considering personal driving forces in the conceptual framework may have helped to shed light on this driving force.

Overall, interconnections between the driving forces of different categories were recognized, especially between economic and political driving forces. For example, policies to reduce water losses (political driving force) were related to productivity increases (economic driving force) and the goal to foster the competitiveness of irrigated agriculture (economic driving force), and they were fostered through financial incentives (political driving force). As Mohr et al. (2023) show, the impact of driving forces on farm change can largely depend on how driving forces co-occur.

## Irrigation-related landscape impact and sustainability outcomes

The irrigation development under study contributed to landscape changes, such as increased field size and a decrease in structural landscape elements. These landscape changes were related to the land consolidation that was tied to the establishment of the UPN. Land consolidations have also been associated with the removal of structural landscape elements in other studies (Jan Benthem 1969; Baudry et al. 2000; Papanastasis et al. 2009; Denac & Kmecl 2021). Nonetheless, certain positive effects for land use sustainability such as higher input efficiency or contribution to rural development have been attributed to land consolidations (Moravcová et al. 2017; Duan et al. 2021). It was further shown that land consolidations serve as an important building stone for the modernization for large-scale irrigation systems (Playán et al. 2024), leading to higher efficiency, productivity, and satisfaction of users (Arslan et al. 2024). These positive effects are largely reflected in the economic/social sustainability results of our study. However, the landscape analysis also showed the drastic changes that the development of the large-scale irrigation system at the SMP site had on the structure of the landscape. These changes are particularly noteworthy when compared to the much smaller landscape change at the SMI site, which was generally influenced by similar driving forces as SMP but did not experience modernization of the irrigation system during the study period.

The loss in structural diversity recognized in the landscape mapping aligns with the loss and fragmentation of habitat mentioned by the environmental experts interviewed. This indicates a negative impact on ecological sustainability as in agricultural landscapes, structural complexity and small parcels are highly relevant for biodiversity (Tscharntke et al. 2021). Regarding water use efficiency, the results in the environmental sustainability dimension are supported by other studies. Water use efficiency improvements with a shift from flood to sprinkler irrigation have been recognized in various regions (Fernández García et al. 2013; Carrillo-Cobo et al. 2014; Eldeiry et al. 2016), as have associated rebound effects, such as e.g. the use of more water-intensive crops, leading to higher overall water consumption (Lecina et al. 2010; Lopez-Gunn et al. 2012; González-Cebollada 2015; Tarjuelo et al. 2015). Water use efficiency increases are hence not necessarily a positive sustainability outcome. Research additionally points to major energy use increases with changes to pressurized irrigation (Jlassi et al. 2016; Berbel et al. 2019; Khadra & Sagardoy 2019). Given the increasing need for irrigation in the face of climate change (Elliott et al. 2014; Fader et al. 2016; FAO 2020), it is essential for policy makers not only to consider the opportunities of irrigation systems, but also to identify their negative externalities and provide ways to reduce them. Socially, the positive outcomes fit with research that rated irrigation development as a good social investment (Borrego-Marín & Berbel 2019). In the economic sustainability dimension, there seems to be a scientific consensus about pressurized piped irrigation networks enabling productivity increases (González-Cebollada 2015; Tarjuelo et al. 2015; Ahmad & Khan 2017; Berbel et al. 2019; Hoffmann & Villamayor-Tomas 2023), and the farmers in this study also experienced significant productivity increases. However, farmers simultaneously experienced increases in energy costs. A high energy cost dependence is of concern, as it has been attributed to increased economic vulnerability of farmers (Sanchis-Ibor et al. 2021).

## Methodological considerations

Overall, the comparative approach applied in this study allowed us to answer the research questions. However, a few methodological limitations need to be pointed out. Regarding the landscape mapping, the difference in spatial resolution of the aerial photographs from 2002 and 2017 may have impacted the comparability of the results. This limitation is estimated to be minor, however, as the resolution of the aerial photographs was sufficient to map the intended landscape structures. A limitation of the qualitative methodology is that the interviewees were mainly found through pre-existing contacts and snowball sampling. This may have led to a selection of interviewees with somewhat similar perspectives (Kirchherr & Charles 2018). Furthermore, while the focus on the perceived driving forces allowed us to get insight into the stakeholders’ perspectives, driving forces may have been missed that different approaches could have captured (Kizos et al. 2018). Similarly, the sustainability outcomes are primarily based on stakeholder perception and would need a more complete assessment, including further quantitative data, for in-depth insights (e.g., Antunes et al. 2017).

## Conclusion

This study provided insight into the irrigation development at two study sites in northern Spain. The development of the large-scale underground piped irrigation system (UPN) was in the interest of diverse actors and was influenced by driving forces across multiple scales and categories. While the interviewees perceived institutional and social driving forces as key influences on the development, technological driving forces were especially rare in the material gathered. Many driving forces were interrelated, with the link between political and economic driving forces being especially evident. To gain a more complete understanding of the driving forces contributing to the irrigation development, future studies may benefit from incorporating other source types such as protocols of institutions involved.

The UPN establishment had a visible impact on landscape development, particularly in terms of a decrease in landscape elements and an increase in field size. The establishment of the UPN also affected the water bodies available in the landscape and the associated species. As such landscape changes can have a negative impacts on ecological sustainability, e.g. biodiversity loss, it is important to consider this aspect in regional planning processes and policy making. For future UPN developments, we thus recommend focusing on preserving semi-natural habitats and the structural diversity of the agricultural landscape. A shift from a focus on water use efficiency increases towards evapotranspiration management and water-efficient crops is also recommended. In future projects, the impact of rising energy costs on farmers should be taken into account. To increase the economic security of farmers, a new funding scheme seems necessary.

The plans for future UPNs that were recognized in this study point to the relevance of scientifically accompanying such large-scale irrigation development. Further studies on sustainability outcomes are pivotal to gain more detailed knowledge about the impact of the development. In particular, information on the perspectives of different stakeholders and regular consulting with farmers is needed in order to incorporate these perspectives into future planning and policy processes of irrigation development.

## Supplementary Information

Below is the link to the electronic supplementary material.

Supplementary file1 (DOCX 851 kb)

## Data Availability

No datasets were generated or analysed during the current study.
