# Supplementary material for: Agricultural irrigation development in Castilla y León (Spain): driving forces and outcomes for landscape and sustainability in the 21st century
Source: Landsc Ecol. 2024 Nov 7;39(11):193. doi: 10.1007/s10980-024-01977-y (PMC11543776; doi:10.1007/s10980-024-01977-y)
Supplement: Supplementary file 1 — Supplementary file1 (DOCX 851 kb) [file 10980_2024_1977_MOESM1_ESM.docx]

Supplementary Information

**Land-use Types**


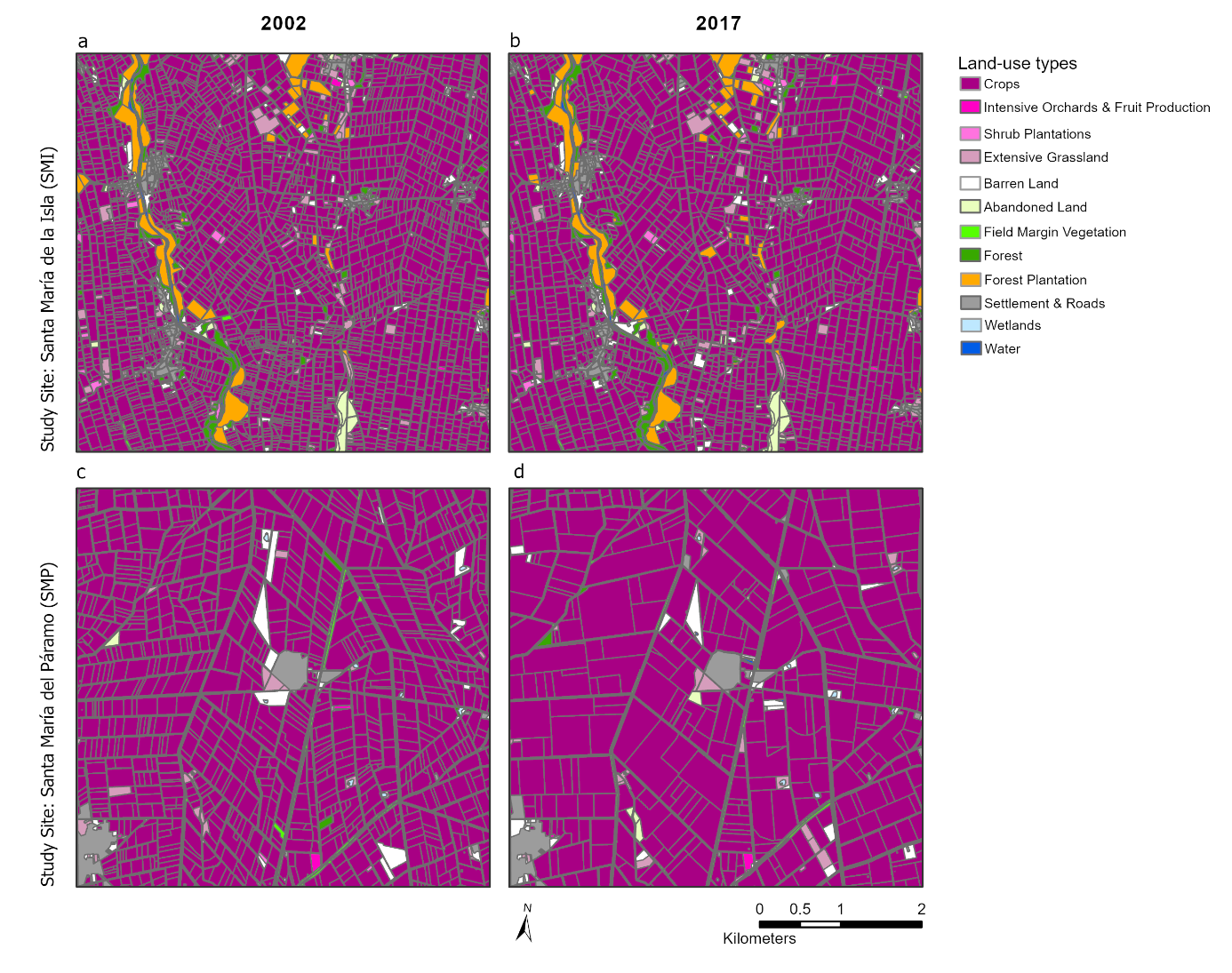


*Fig. 9: Land-use types in the two study sites in 2002 and 2017. Each study site covers 25 km^2^.*

*Table 2: Area covered by each land-use type (rounded to whole numbers) in the two study sites Santa María del Páramo (SMP) and Santa María de la Isla (SMI) in 2002 and 2017.*

| Land-use type | Area covered by land-use type [ha] | | | |
| --- | --- | --- | --- | --- |
|  | Value SMI 2002 | Value SMI 2017 | Value SMP 2002 | Value SMP 2017 |
| Crops | 2030 | 2014 | 2130 | 2201 |
| Intensive Orchards & Fruit Production | 1 | 3 | 3 | 2 |
| Shrub Plantations | 9 | 8 | 1 | 0 |
| Extensive Grassland | 66 | 53 | 19 | 25 |
| Field Margin Vegetation | 48 | 47 | 165 | 108 |
| Forest Plantation | 86 | 97 | 0 | 0 |
| Barren Land | 23 | 32 | 46 | 36 |
| Abandoned Land | 19 | 23 | 2 | 6 |
| Forest | 57 | 57 | 4 | 3 |
| Settlement & Roads | 143 | 148 | 125 | 115 |
| Wetlands | 0 | 0 | 3 | 2 |
| Water | 18 | 18 | 2 | 2 |
